# Chronic Kidney Disease Severity Is Associated With Selective Expansion of a Distinctive Intermediate Monocyte Subpopulation

**DOI:** 10.3389/fimmu.2018.02845

**Published:** 2018-12-06

**Authors:** Serika D. Naicker, Sarah Cormican, Tomás P. Griffin, Silvia Maretto, William P. Martin, John P. Ferguson, Deirdre Cotter, Eanna P. Connaughton, M. Conall Dennedy, Matthew D. Griffin

**Affiliations:** ^1^CÚRAM Centre for Research in Medical Devices, School of Medicine, Regenerative Medicine Institute (REMEDI), College of Medicine, Nursing and Health Sciences, National University of Ireland, Galway, Ireland; ^2^Nephrology Services, Saolta University Healthcare Group, Galway, Ireland; ^3^Centre for Diabetes, Endocrinology and Metabolism, Galway University Hospitals, Saolta University Healthcare Group, Galway, Ireland; ^4^HRB Clinical Research Facility, National University of Ireland, Galway, Ireland

**Keywords:** monocytes, chronic kidney disease, inflammation, neutrophils, HLA-DR, eGFR

## Abstract

Chronic kidney disease (CKD) affects 11–13% of the world's population and greatly increases risk of atherosclerotic cardiovascular disease (ASCVD) and death. It is characterized by systemic inflammation and disturbances in the blood leukocytes that remain incompletely understood. In particular, abnormalities in the numbers and relative proportions of the three major monocyte subsets—classical, intermediate, and non-classical—are described in CKD and end-stage renal disease. In this study, we characterized absolute numbers of blood leukocyte subtypes in adults with renal function varying from normal to advanced CKD. The primary aim was to identify monocyte subpopulations that associated most closely with current estimated glomerular filtration rate (eGFR) and subsequent rate of eGFR decline. Leucocyte and monocyte populations were enumerated by multi-color flow cytometry of whole blood and peripheral blood mononuclear cell (PBMC) samples from adults with CKD stage 1–5 (*n* = 154) and healthy adults (*n* = 33). Multiple-linear regression analyses were performed to identify associations between numbers of leucocyte and monocyte populations and clinical characteristics including eGFR and rate of eGFR decline with adjustment for age and gender. In whole blood, total monocyte and neutrophil, but not lymphocyte, numbers were higher in adults with CKD 1-5 compared to no CKD and were significantly associated with current eGFR even following correction for age. In PBMC, classical and intermediate monocyte numbers were higher in CKD 1-5 but only intermediate monocyte numbers were significantly associated with current eGFR in an age-corrected analysis. When intermediate monocytes were further sub-divided into those with mid- and high-level expression of class II MHC (HLA-DR^mid^ and HLA-DR^hi^ intermediate monocytes) it was found that only DR^hi^ intermediate monocytes were increased in number in CKD 1-5 compared to no CKD and were significantly associated with eGFR independently of age among the total (No CKD + CKD 1-5) study cohort as well as those with established CKD (CKD 1-5 only). Furthermore, blood number of DR^hi^ intermediate monocytes alone proved to be significantly associated with subsequent rate of renal functional decline. Together, our data confirm neutrophil and monocyte subset dysregulation in CKD and identify a distinct subpopulation of intermediate monocytes that is associated with higher rate of loss of kidney function.

## Introduction

The term chronic kidney disease (CKD) describes the adverse effect of heterogeneous disorders on kidney structure and function (1) and has a rising worldwide prevalence of between 11 and 13% (2). Estimated global mortality associated with CKD has increased by 32% from 2005 to 2015 (3). Reduction in glomerular filtrate rate (GFR), a hallmark of CKD, is associated with substantially increased mortality risk due to atherosclerotic cardiovascular disease (ASCVD) (4–7). For the delivery of effective healthcare to those with CKD, there is a significant unmet need for strategies to more accurately predict risk of declining renal function, guide therapeutic decisions and categorize clinical trial subjects (8).

Chronic kidney disease results in abnormalities of multiple physiological processes including removal of waste products of metabolism, electrolyte balance, blood pressure homeostasis, and endocrine pathways which may contribute to ASCVD (1, 9–12). Conventional ASCVD risk factors prevalent in CKD are managed by well-established methods including medication to control blood pressure, lipid parameters, and glycemia. Other non-conventional ASCVD risk factors in CKD such as chronic inflammation do not yet have definitive treatments. High numbers of circulating leukocytes, primarily monocytes, and persistent low-grade inflammation may be driven by accumulation of uremic toxins, impaired cytokine excretion, and other factors (13, 14). Circulating monocytes may differentiate into lipid-laden macrophages and contribute to atherosclerotic lesions, plaque formation, and fibrosis (15). The responses of blood vessels and surrounding tissues to circulating monocytes and their mediators are involved in kidney disease-associated endothelial dysfunction, calcification, and atherogenesis (16, 17). Consistent with this, elevated blood monocyte numbers have been reported to correlate with renal function, ASCVD and risk of progression to end-stage renal disease (ESRD) (18, 19).

Monocytes are a major cellular component of the innate immune system and have been recently implicated in complications and progression of CKD (15, 19). This is inherently plausible given their characteristic ability to rapidly transmigrate from the bloodstream to sites of inflammation and their potent responses to inflammatory stimuli (20). Recent advances have revealed substantial phenotypic and functional heterogeneity among circulating human monocytes which may be relevant to CKD pathogenesis (20–23). Monocytes express specific cytokines and adhesion molecules that are central to the pathogenesis of CKD-associated inflammation. Cytokines promote activation and trans-endothelial migration of immune effector cells leading to a chronic pro-fibrotic inflammatory infiltration of the kidneys and other tissues (24–26). Three monocyte subsets are currently recognized on the basis of surface expression of CD14 and CD16: classical (CD14^++^CD16^−^), intermediate (CD14^++^CD16^+^), and non-classical (CD14^+^CD16^++^). These subsets have been documented to have a range of distinct phenotypic and functional features (27). Alterations to the circulating monocyte repertoire have been reported in CKD by several groups, with higher numbers of CD16^+^ monocytes being specifically linked to worse cardiovascular outcomes (28–33). A pathophysiological link between intermediate monocytes and accelerated atherosclerosis is suggested by the avidity of this monocyte subset for modified lipoprotein scavenging and cholesterol efflux (32, 34).

While studies related to the ability of monocytes to predict renal functional decline are limited, intermediate monocyte numbers have been inversely correlated with estimated GFR (eGFR) (19, 31, 35). Furthermore, current understanding of the origin and *in vivo* function of intermediate monocytes and their relationship to classical and non-classical monocytes is incomplete. Microarray analysis identified 1,554 genes common to all monocyte subsets but only 135 were highly expressed by intermediate monocytes, many of which are involved in activation, angiogenesis, oxidative stress, antigen processing and antigen presentation (36, 37). Recent experimental evidence from healthy humans indicates that a linear developmental relationship exists among the three subsets but the details of how this process may be altered in disease settings are not known (23). Recently, our group has demonstrated a further subdivision of intermediate monocytes based on surface expression of the class II human leukocyte antigen, HLA-DR. In healthy adults and adults with obesity, we observed the presence of sub-populations within the intermediate gate that exhibited, respectively, mid- and high-level expression of HLA-DR—termed DR^mid^ and DR^hi^ intermediate monocytes (22). Functional and phenotypic differences between the two intermediate monocyte subpopulations included differential expression of chemokine and adhesion receptors (with DR^mid^ more closely resembling classical monocytes and DR^hi^ more closely resembling non-classical monocytes) and differential uptake rates of oxidized and acetylated low-density lipoproteins (LDL) (with DR^mid^ having higher uptake than DR^hi^). In obese compared with non-obese adults, we observed increased numbers of intermediate monocytes in blood that was entirely due to relative expansion of the DR^mid^ (and not the DR^hi^) subpopulation (22).

In this study, we aimed to evaluate the major circulating leukocyte populations, the conventionally-defined blood monocyte subsets and the recently-described intermediate monocyte subpopulations in blood samples from adults with non-renal replacement therapy (RRT)-requiring CKD of varying severity. We also sought to determine whether circulating numbers of the leukocyte subpopulations studied have potential predictive value for subsequent rate of decline of eGFR in CKD.

## Materials and Methods

### Ethical Considerations

The study was carried out in accordance with the ethical principles of the Declaration of Helsinki and the International Conference on Harmonisation's Good Clinical Practice Guidelines (ICH GCP) with written informed consent from all subjects. All subjects gave written informed consent in accordance with the Declaration of Helsinki. The protocol was approved by the Galway University Hospitals (GUH) Clinical Research Ethics Committee and the National University of Ireland Galway (NUI Galway) Research Ethics Committee.

### Study Design

Adults with CKD stages 1 to 5 were enrolled by informed consent from nephrology outpatient clinics at GUH between January 2014 and May 2016. Stages of CKD were defined using the modified (4-parameter) Modification of Diet in Renal Disease (MDRD) equation as routinely reported by the GUH Clinical Biochemistry Laboratory during the time-period covered by the study (38). Inclusion criteria for the CKD 1-5 cohort were: (a) Age ≥ 18 years, (b) Diagnosis of CKD based on eGFR, urinalysis and clinical manifestations, (c) Not currently being treated for infection, cancer, acute cardiovascular event or hematological condition other than anemia, (d) Willing and able to provide informed consent, (e) Hemoglobin level ≥ 10 g/dL, (f) Not currently receiving chronic hemodialysis or peritoneal dialysis, (g) Not the recipient of a kidney transplant, (h) Not known to be positive for human immunodeficiency virus, hepatitis B virus or hepatitis C virus, (i) Not currently receiving immunosuppressive therapy.

Healthy volunteers (subsequently referred to as no CKD) were enrolled at NUI Galway and at GUH. Inclusion criteria for the no CKD cohort were as follows: (a) Age ≥ 18 years, (b) No active medical conditions, (c) Not taking any medication (over the counter or prescription) within 48 h of study (excluding contraceptives), (d) Willing and able to provide informed consent, (e) No current or past history of a kidney disease.

Clinical, laboratory and radiological data were recorded in a secure, password-protected, web-based clinical database (Distiller^®;^, SlidePath, Dublin, Ireland), developed in collaboration with the Health Research Board (HRB) Clinical Research Facility Galway. Paper and electronic medical records of the enrolled study subjects were reviewed by medically-qualified members of the research team and relevant fields were compiled in the database. Longitudinal changes in renal function were assessed using eGFR values from date of enrolment to most recent renal function values available on September 30th 2017. Serial laboratory results were extracted from the eMED*Renal* clinical data system (Mediqal H.I., Aston, UK).

### Blood Sample Collection, Processing, and Total Cell Counts

Freshly-drawn peripheral venous blood samples were collected in 10 mL Ethylenediaminetetraacetic acid (EDTA) Vacutainer® tubes (BD Medical Supplies, Crawley, UK). Blood sample collection was performed once for each participant at time of consent. Samples were transported at 4°C and processed within 2 h of collection. For enumeration of the major subtypes of peripheral blood leukocytes (PBL), 100 μL of anti-coagulated blood collected in EDTA vacutainers were incubated with mouse anti-human CD45-APC (BD Biosciences, San Jose, CA) and mouse anti-human CD14-PerCP-Cy5.5 (Miltenyi Biotec, Cologne, Germany). Red blood cell (RBC) lysis was performed using RBC lysis buffer (BD Biosciences) according to the manufacturer's instructions. The remaining cells were washed and re-suspended in 200 μL of Ca^2+/^Mg^2+^-free Dulbecco's phosphate-buffered saline (PBS) (Gibco-Life Technologies, Carlsbad, CA). Total cell number was recorded by timed acquisition of events per unit volume using an Accuri^TM^ C6 flow cytometer (BD Biosciences, San Jose, CA, USA). Compensation and gate settings were generated using single stain controls. Data files were analyzed using BD Accuri^TM^ C6 CFlow Sampler Software (BD Biosciences). The absolute numbers of circulating neutrophils, monocytes, and lymphocytes were calculated and expressed as cells per mL of whole blood from the proportion of total cells recorded within predetermined gates based on forward vs. side scatter profiles and surface staining profiles.

### Peripheral Blood Mononuclear Cell Isolation and Multi-Color Flow Cytometry Analysis

Isolation of peripheral blood mononuclear cells (PBMC) was performed by layering 3 mL aliquots of EDTA-anti-coagulated blood over 3 mL of endotoxin-free Ficoll-Plaque Plus® medium (GE Healthcare, Little Chalfont, U.K.) in 15 mL Falcon tubes (Sarstedt, Nümberbrecht, Germany). Samples were centrifuged at 420 relative centrifugal force (RCF) for 22 min at 4°C with break. The visible cloudy, “buffy coat” layer of PBMC at the interface of the plasma and RBC layers was carefully removed using a 3 mL plastic Pasteur pipette (Starstedt). The PBMC were transferred to a new 15 mL Falcon tube (Sarstedt) and made up to 10 mL with fluorescent activating cell sorting (FACS) buffer [2% fetal calf serum (Lonza, Basel, Switzerland), PBS and 0.05% NaN_3_ (Sigma-Aldrich, St. Louis, MO, USA)]. The PBMC were pelleted by centrifugation at 300 RCF for 10 min at 4°C. Next, the supernatant was discarded, the PBMC pellet was re-suspended in 5 mL FACS buffer and the wash step was repeated using the same protocol as described above. Afterwards, the PBMC pellet was re-suspended in 1 mL FACS buffer and counted using a hemocytometer. This protocol for PBMC isolation for the purpose of monocyte subpopulation analysis has been previously optimized (22) and shown to provide consistent results when all steps are performed at 4°C (EP Connaughton, MC Dennedy and MD Griffin, unpublished results). Aliquots containing 5 × 10^5^ cells were transferred to each 3 mL polystyrene FACS tube (Sarstedt) and made up to a final volume of 100 μL with FACS buffer per tube.

The PBMC aliquots were incubated with optimized volumes of fluorochrome-conjugated monoclonal antibodies [mouse anti-human CD45-V500, mouse anti-human CD16-FITC, mouse anti-human HLA-DR-APC, mouse anti-human CD56-V450 (BD Biosciences), mouse anti-human CD14-PerCP-Cy5.5, mouse anti-human CD33 APC-Vio770 (Miltenyi Biotec), and mouse anti-human CX2CR1-Pe-Cy7 (Biolegend, San Diego CA, USA)] for 20 min at 4°C to allow identification of the three conventional human monocyte subsets and the newly identified intermediate monocyte subpopulations (22). Each aliquot of PBMC was washed with 1 mL FACS buffer, centrifuged at 300 RCF for 5 min at 4°C and finally re-suspended in 200 μL FAC buffer. Flow cytometric analysis was performed using a BD FACS Canto™ II flow cytometer (BD Biosciences) which was calibrated according to the manufacturer's recommendations. Fluorescence compensation was set using single-stained controls and matching median compensation algorithms were applied. Samples were processed within 2 h of collection and dead cells were excluded using FSC-A vs. SSC-A gating by flow cytometry. Optimisation studies demonstrated consistently high viability of gated populations using this gating approach (SD Naicker, EP Connaughton, MC Dennedy and MD Griffin, unpublished results). Fluorescence minus one (FMO) controls were used to set analysis gates controls and data were analyzed using Diva v8.0.1 acquisition software (BD Biosciences) or FlowJo^®;^ 7.6.5 software (TreeStar Inc., Olten, Switzerland).

### Calculation of Annual Rate of Renal Function Decline

To determine the annual rate of renal functional decline, the slope of best fit was calculated for each study subject by linear regression of longitudinal eGFR measurements recorded in the medical records between the date of study entry and September 2017. For subjects who died or began RRT during the study observation period, linear regression analysis was halted at the last measurement prior to initiation of RRT or death. Participants with fewer than three eGFR values during the observation period, those who commenced RRT within 1 month of enrolment and those whose serial eGFR values clearly clustered around episodes of acute kidney injury (AKI) were excluded from the analyses of rate of renal functional decline.

### Statistical Analyses

Statistical analyses were performed using GraphPad® Prism Version 6 (GraphPad Software, CA, USA), International Business Machines Corporation (IBM) Statistical Package for the Social Science (SPSS) version 23 (IBM, Armonk, NY,.S.A) and Minitab Version 18® (Minitab Inc., State College, Penn., USA). Continuous data were represented using means (standard deviation) where normally distributed and medians (interquartile range [IQR]) when non-normally distributed. Comparisons for non-parametric data between participants with no CKD and CKD 1-5 were performed using Mann-Whitney *U*-test. Categorical data were summarized as frequencies (percentages). Fisher's exact test was used to compare differences in proportions between the groups. The relationship between rate of eGFR decline and the different monocyte subpopulations was explored using Spearman's correlation. Multiple linear regression models, using standardized coefficients (95% confidence interval) were used to explore the relationships between the response variable (renal function [eGFR]) and the explanatory variables (clinical demographics and different leucocyte populations). The standardized coefficient is the change in eGFR per standard deviation (for parametric data) and IQR (for non-parametric data) increase in the explanatory variable. For the first model (Model a) for each analysis, all variables of interest were included. In the subsequent model (Model b), non-significant variables were excluded. Age was significantly associated with eGFR in all Model a analyses. It was, therefore, included as an explanatory variable in all Model b analyses, thus allowing for its influence on the associations between immune cell counts and eGFR to be accounted for. For all analyses performed, statistical significance was assigned to *P*-values < 0.05.

## Results

A total of 187 subjects was included in the study −33 healthy volunteers with no CKD and 154 outpatients with CKD 1–5. Compared to no CKD, those with CKD 1–5 were older and had predictable differences in serum creatinine and urea concentrations (Table 1). The mean eGFR for the CKD 1–5 group was 39 ± 22 mL/min/1.73 m^2^. The documented causes of CKD and co-morbidities of the CKD 1–5 cohort are summarized in Table 2. A range of CKD etiologies was documented of which the most frequent were unknown cause, diabetes mellitus, and glomerulonephritis. Frequent co-morbidities included hypertension, diabetes mellitus, bone and joint disease, and ASCVD.

**Table 1:** Relevant basic demographic and renal functional indices of study cohort.

	**No CKD**	**CKD 1-5^†^**	**P^‡^**
Number (*n*)	33	154	–
^ 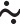 ^Gender [F]	16 (48.5)	61 (39.6)	0.436
^§^ Age (yrs)	40 (26.0–52.0)	66 (48.0–75.0)	< 0.001
^§^ Serum creatinine (μmol/L)	77 (71–83)	161 (117–224)	< 0.001
^§^ Serum urea (mmol/L)	4.0 (3.0–5.0)	11.0 (7.0–16.0)	< 0.001
^§^ MDRD eGFR (mL/min/1.73 m^2^)	86 (78–99)	33 (23–50)	< 0.001

† *CKD, chronic kidney disease; MDRD eGFR, estimated glomerular filtration rate, 4-parameter MDRD equation*.

‡ *P-value represents the comparison between no CKD and CKD as determined by Fishers Exact test for categorical data by Mann-Whitney two-tailed t-test for non-parametric data*.

^
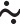
^*Number (%)*,

§ *Median (IQR)*.

**Table 2:** Documented CKD etiologies and co-morbidity profiles of CKD 1-5 study cohort.

	**Number (Total = 154)**	**% of Total**
**DOCUMENTED CKD ETIOLOGY**		
Unknown	34	22.1
Diabetes Mellitus	29	18.8
Glomerulonephritis	25	16.2
Other diagnosis	25	16.2
Hypertension	15	9.7
Polycystic kidney disease	7	4.5
Other congenital renal/urological disease	6	3.9
Interstitial nephritis	5	3.2
Obstructive nephropathy	4	2.6
Other hereditary disease	3	1.9
Chronic infection	1	0.6
^*^CO-MORBIDITIES		
Hypertension	123	79.9
Diabetes Mellitus	43	27.9
Diabetes Mellitus Type 1	6	3.9
Diabetes Mellitus Type 2	37	24.0
Bone and Joint Disease	23	14.9
Coronary Artery Disease	20	13.0
Peripheral/Aortic Vascular Disease	20	13.0
Chronic Lung Disease	18	11.7
Autoimmune Disease	16	10.4
Atrial Fibrillation	14	19.1
Heart Failure/ Cardiac Valve Disease	9	5.8
Chronic Liver Disease	1	0.7

* *Participants may have more than 1 co-morbidity*.

### Blood Monocyte and Neutrophil Numbers Are Increased in CKD 1–5 and Are Independently Associated With eGFR

**Figure 1:**
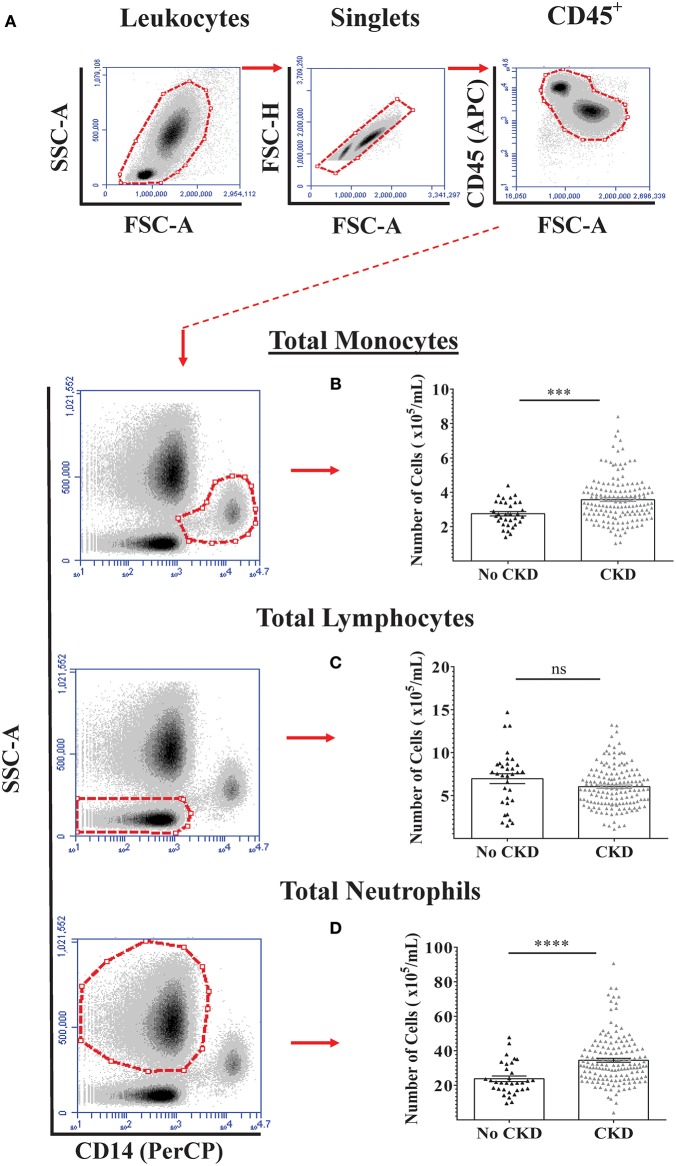
Circulating PBL numbers in healthy adults and adults with varying stages of CKD. Absolute numbers of the three major PBL subtypes were quantified by flow cytometry in blood samples from 187 adults with No CKD (*n* = 33) and CKD 1-5 (*n* = 154). **(A)** Specific CD45^+^ PBL subpopulations were identified based on side scatter characteristics and surface expression of CD14. Representative gating strategy used for the identification and quantification of **(B)** monocyte, **(C)** lymphocyte, and **(D)** neutrophil numbers per mL of whole blood. Graphs illustrate the median, IQR, and upper and lower limit for each leukocyte subtype for No CKD and CKD groups (box and whisker plots) with data-points for individual subjects superimposed (triangles). Statistical comparisons performed using Mann-Whitney U-test. ns *p* > 0.05, ***p < 0.001, ****p < 0.0001.

Flow cytometry analysis was initially used to quantify total numbers of monocytes, lymphocytes, and neutrophils in freshly-drawn whole blood samples from the total study cohort of 187 subjects and the results for no CKD were compared to those for CKD 1–5 (Figures 1A–D). As shown, total blood monocyte and neutrophil numbers were higher for CKD 1–5 while blood lymphocyte numbers were similar for the two groups. To demonstrate the relationships between PBL numbers and renal function (eGFR) within the whole study cohort, separate multiple linear regressions were applied for the variables of age, gender, and total blood numbers of monocytes, lymphocytes, and neutrophils (Table 3, Model 1a). This indicated significant associations between current eGFR and age, total monocyte, and neutrophil counts, but not gender and total lymphocyte count. As age was, predictably, associated with eGFR (*P* < 0.001), multiple regression with age-adjustment was then performed (Table 3, Model 1b). This confirmed age-independent associations of blood monocyte number (*P* = 0.03) and blood neutrophil number (*P* = 0.016) with eGFR.

**Table 3:** Multiple linear regression models to determine the relationships between demographic indices, circulating leukocyte populations, and renal function in study subjects with No CKD + CKD 1-5.

**Model**	**Cohort**	**Number**	**Response variable**	**Explanatory variable**	**Coefficient**	**95% CI**	**^†^*P***
1a	No CKD + CKD 1-5	187	eGFR	Constant	110.22	94.22, 126.21	< 0.001
				Age (years)	−26.01	−31.84, −20.17	< 0.001
				Gender (M)	2.55	−9.14, −0.98	0.46
				^#^**Monocyte**	−5.27	−9.47, −1.06	**0.015**
				^#^Lymphocyte	2.83	−1.93, 7.59	0.24
				^#^**Neutrophil**	−5.06	−9.14, −0.98	**0.015**
1b (ageadjusted)	No CKD + CKD 1-5	187	eGFR	Constant	115.83	102.38, 129.28	< 0.001
				Age (years)	−26.64	−32.36, −20.92	< 0.001
				^#^**Monocyte**	−4.49	−8.53, −0.44	**0.03**
				^#^**Neutrophil**	−5.01	−9.08, −0.94	**0.02**

† *Statistical test = a) Multiple linear Regression Model, b) Multiple linear Regression Model with age correction*.

# *Represented as cells/mL. Cell types for which significant results were observed are bolded for emphasis*.

### Blood Intermediate Monocyte Numbers Are Most Closely Associated With Renal Function

**Figure 2:**
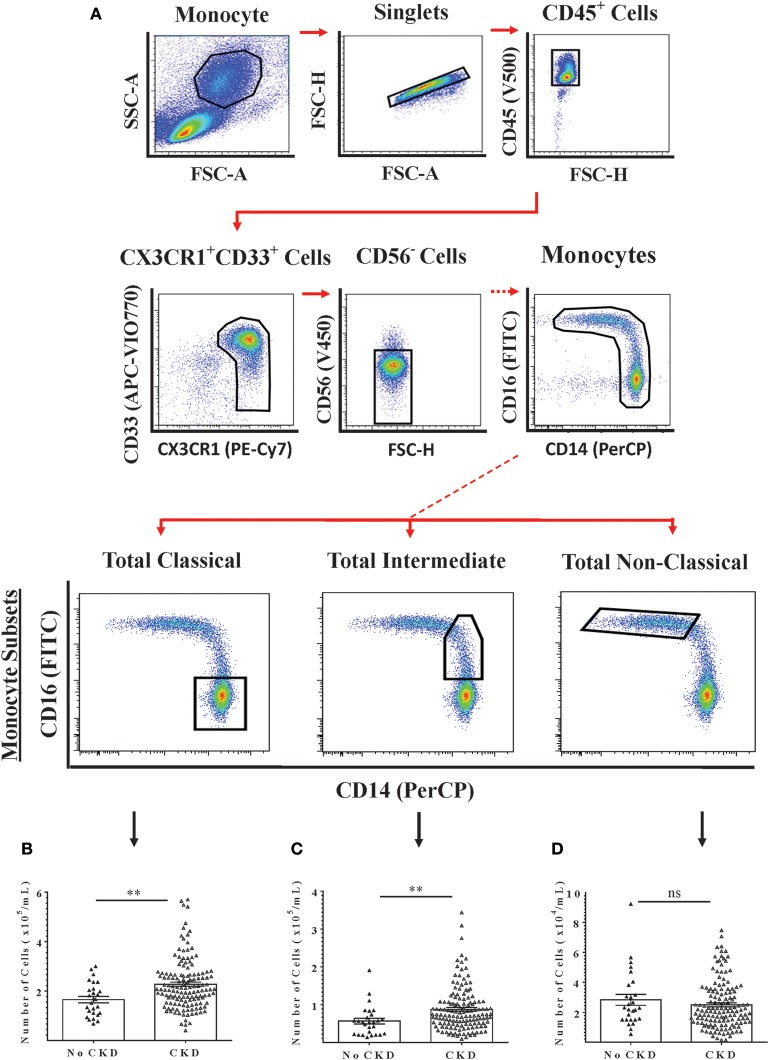
Multi-color flow cytometry for the identification and quantification of total circulating monocyte subsets. Monocyte subsets were identified using a 6-color staining strategy in freshly-isolated PMBC from 169 adults with No CKD (*n* = 27) or CKD 1–5 (*n* = 142). **(A)** Representative dot plots are shown to illustrate the gating strategy for the identification and quantification of total monocytes (Upper Panels) and of the three currently-recognized monocyte subsets. **(B–D)** Graphs are shown of absolute numbers of classical, intermediate, and non-classical monocytes respectively expressed as cells per mL of whole blood for the two groups. Graphs illustrate the median, IQR, and upper and lower limit for each leukocyte subtype for No CKD and CKD groups (box and whisker plots) with data-points for individual subjects superimposed (triangles). Statistical comparisons performed using Mann-Whitney U-test. ns *p* > 0.05, **p < 0.01.

The relationships between renal function and circulating numbers of the three monocyte subsets that are currently recognized by convention were next determined within the study cohort. Freshly-isolated PBMC from 169 subjects (27 with no CKD and 142 with CKD 1–5) were analyzed by flow cytometry using a 6-color analysis strategy for absolute numbers of classical, intermediate and non-classical monocytes (Figure 2A) (22). From these analyses, absolute numbers of each monocyte subset per unit volume of blood were calculated. As shown in Figures 2B–D, circulating numbers of classical and intermediate monocytes were higher for CKD 1–5 compared to no CKD while non-classical monocytes numbers were not different. Multiple linear regression models for all subjects included in the PBMC analysis indicated significant associations with eGFR for age, classical monocyte number, and intermediate monocyte number but not for gender and non-classical monocyte number (Table 4, Model 2a). This association with renal function was stronger for intermediate than for classical monocytes (*P* = 0.009 vs. *P* = 0.049). The association between intermediate, but not classical monocytes, and eGFR remained significant in an age-adjusted model (*P* = 0.030) (Table 4, Model 2b).

**Table 4:** Multiple linear regression models to determine the relationships between demographic indices, currently-recognized monocyte subsets, and renal function in study subjects with no CKD + CKD 1-5.

**Model**	**Cohort**	**Number**	**Response variable**	**Explanatory variable**	**Coefficient**	**95% CI**	**^†^*P***
2a	No CKD + CKD 1-5	169	eGFR	Constant	100.54	86.88, 114.2	< 0.001
				Age (years)	−25.39	−31.38, −19.4	< 0.001
				Gender (M)	5.11	−1.97, 12.18	0.16
				^#^**Classical**	−4.18	−8.34, −0.02	**0.049**
				^#^**Intermediate**	−5.36	−9.35, −1.37	**0.009**
				^#^Non-classical	3.31	−1.07, 7.69	0.14
2b (age adjusted)	No CKD + CKD 1-5	169	eGFR	Constant	105.38	92.43, 118.32	< 0.001
				Age (years)	−25.06	−31.07, −19.08	< 0.001
				^#^Classical	−3.98	−8.15, 0.19	0.06
				^#^**Intermediate**	−4.26	−8.11, −0.41	**0.03**

† *Statistical test = a) Multiple linear Regression Model, b) Multiple linear Regression Model with age correction*.

# *Represented as cells/mL. Cell types for which significant results were observed are bolded for emphasis*.

### A Distinct, HLA-DR^hi^ Intermediate Monocyte Subpopulation Is Numerically Increased in the Peripheral Blood of Patients With CKD Stages 1–5

**Figure 3:**
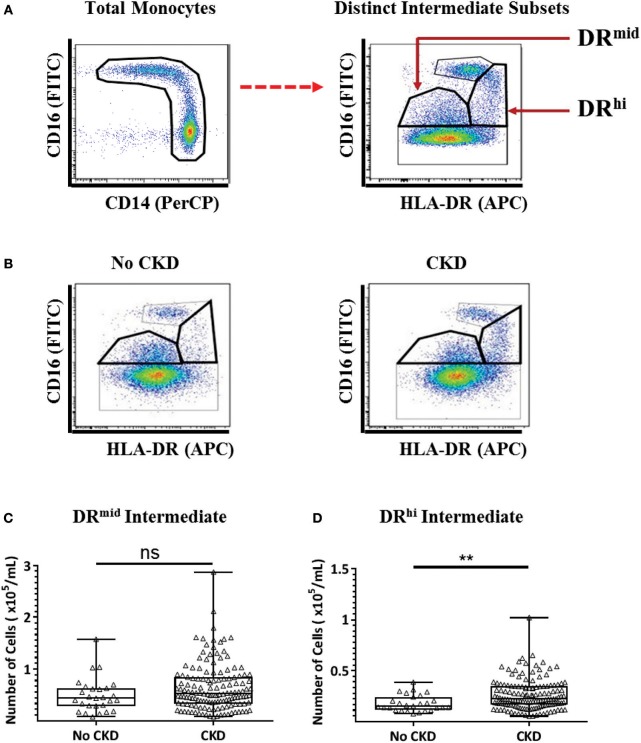
Multi-color flow cytometry for the identification and quantification of distinct DR^mid^ and DR^hi^ intermediate monocyte subpopulations. **(A)** Representative example of the gating strategy used for the identification and quantification of two distinct intermediate monocyte subpopulations based on HLA-DR surface expression. **(B)** Representative examples of flow cytometry dot plots from subjects without (no CKD) and with (CKD) chronic kidney disease showing the distribution of four monocyte subpopulations, **(C,D)** Graphs are shown of absolute numbers of DR^mid^ **(C)** and DR^hi^ **(D)** intermediate monocytes expressed as cells per mL of whole blood for study subjects with No CKD (*n* = 27) or CKD 1–5 (*n* = 142). Graphs illustrate the median, IQR, and upper and lower limit for each leukocyte subtype for No CKD and CKD groups (box and whisker plots) with data-points for individual subjects superimposed (triangles) Statistical comparisons performed using Mann-Whitney U-test. ns *p* > 0.05, **p < 0.01.

We recently reported the presence of two distinct subpopulations of intermediate monocytes in the blood of healthy adults and obese adults without diabetes on the basis of mid- and high-level surface expression of HLA-DR (22). The two subpopulations, which we term DR^mid^ and DR^hi^ intermediate monocytes were, respectively, more phenotypically similar to classical and non-classical monocytes. To determine whether CKD is associated with a selective alteration in the intermediate monocyte repertoire, a flow cytometry analysis strategy was applied to the PBMC samples from the same 169 study subjects as for the conventional monocyte analysis (Figure 3A). Representative examples of the gating strategy are shown in Figure 3B to display differences in intermediate monocyte populations between no CKD and CKD populations. As shown in Figures 3C,**D**, the absolute number of DR^hi^ intermediate monocytes was higher in subjects with CKD 1–5 compared to no CKD (*P* = 0.003), while the number of DR^mid^ intermediate monocytes was not (*P* = 0.09).

The results of this alternative analysis of monocyte subpopulations among the no CKD and CKD 1-5 cohort were analyzed with multiple linear regression to explore the relationships with renal function. These analyses indicated that age, DR^hi^ intermediate and non-classical monocyte number but not gender, classical monocyte number and DR^mid^ intermediate monocyte number were significantly associated with eGFR (Table 5, Model 3a). This association with renal function was stronger for DR^hi^ intermediate monocytes (*P* = 0.005) and remained significant (*P* < 0.001) in an age-adjusted model (Table 5, Model 3b). When the regression models were repeated using only data for subjects with CKD 1–5, only DR^hi^ intermediate monocyte number demonstrated initial and age-adjusted associations with eGFR (*P* = 0.025, *P* = 0.03 respectively) (Table 6, Models 4a and 4b). Thus, of the monocyte subsets and subpopulations studied, the DR^hi^ intermediate subpopulation was found to have the most significant relationship with concurrently estimated renal function in analyses that combined healthy volunteers and adults with known CKD as well as separate analyses restricted to CKD subjects only.

**Table 5:** Multiple linear regression models to determine the relationships between demographic indices, alternatively-analyzed monocyte subsets, and renal function in study subjects with no CKD + CKD 1-5.

**Model**	**Cohort**	**Number**	**Response variable**	**Explanatory variable**	**Coefficient**	**95% CI**	**^†^*P***
3a	No CKD + CKD 1-5	169	eGFR	Constant	97.24	83.39, 111.09	< 0.001
				Age (years)	−24.08	−30.13, −18.03	< 0.001
				Gender (M)	6.08	−0.97, 13.14	0.09
				^#^Classical	−2.61	−6.88, 1.66	0.23
				^#^DR^mid^ Int.	−0.23	−4.8, 4.35	0.92
				^#^**DR**^hi^ **Int**.	−9.36	−15.82, −2.91	**0.005**
				**Non-classical**	5.59	0.83, 10.34	**0.02**
3b (age adjusted)	No CKD + CKD 1-5	169	eGFR	Constant	96.84	84.98, 108.71	< 0.001
				Age (years)	−24.65	−30.67, −18.63	< 0.001
				^#^**DR**^hi^ **Int**.	−9.6	−14.9, −4.29	** < 0.001**
				^#^**Non-classical**	5.77	1.05, 10.49	**0.017**

† *Statistical test = a) Multiple linear Regression Model, b) Multiple linear Regression Model with age correction*.

# *Represented as cells/mL. Cell types for which significant results were observed are bolded for emphasis*.

**Table 6:** Multiple linear regression models to determine the relationships between demographic indices, alternatively-analyzed monocyte subsets, and renal function in study subjects with CKD 1–5.

**Model**	**Cohort**	**Number**	**Response variable**	**Explanatory variable**	**Coefficient**	**95% CI**	**^†^*P***
4a	CKD 1-5	142	eGFR	Constant	73.11	58.85, 87.36	< 0.001
				Age (years)	−16.12	−22.2, −10.04	< 0.001
				Gender (M)	4.93	−1.79, 11.68	0.15
				^#^Classical	−0.72	−4.60, 3.16	0.71
				^#^DR^mid^ Int.	0.66	−3.51, 4.83	0.75
				^#^**DR**^hi^ **Int**.	−6.65	−12.45, −0.85	**0.025**
				^#^Non-classical	2.38	−2.36, 7.11	0.32
4b (age adjusted)	CKD 1-5	142	eGFR	Constant	75.75	63.62, 87.88	< 0.001
				Age (years)	−16.18	−22.21, −10.16	< 0.001
				^#^**DR**^hi^ **Int**.	−4.56	−8.76, −0.37	**0.033**

† *Statistical test = a) Multiple linear Regression Model, b) Multiple linear Regression Model with age correction*.

# *Represented as cells/mL. Cell types for which significant results were observed are bolded for emphasis*.

### Blood DR^hi^ Intermediate Monocyte Number Is Associated With Subsequent Rate of Renal Functional Decline

Subsequent to the time-point of study enrolment and blood sampling, the annual rate of renal function decline was calculated over a mean follow-up time of 25.4 ± 9.2 months for 135 of the 154 study subjects with CKD 1–5. The remaining 19 were excluded from the follow-up analysis due to inadequate number of eGFR values (*n* = 13) clustering of renal function measurements around episodes of AKI (*n* = 3) and initiation of haemodialysis within 1 month of enrolment (*n* = 3). Of the 135 for whom subsequent rate of eGFR decline could be calculated, all had baseline whole blood flow cytometry quantification of major PBL subtype and 124 had PBMC flow cytometry quantification of conventional and alternative monocyte subset analysis. To explore the relationship between eGFR decline and the various blood cell populations, multiple linear regression modeling with age correction was applied (Table 7). In the case of the three major PBL types, none were significantly associated with rate of eGFR decline (Table 7, Model 5). A model including the three conventionally defined monocyte subsets also showed no significant relationships between the baseline cell numbers and subsequent eGFR decline rate (Table 7, Model 6). Finally, however, an age-corrected model that included four monocyte subpopulations—classical, DR^mid^ intermediate, DR^hi^ intermediate, and non-classical, revealed a statistically significant association between baseline DR^hi^ intermediate monocyte number and subsequent rate of eGFR decline (Table 7, Model 6). Thus, compared to all other sub-populations, higher DR^hi^ intermediate monocyte number in the blood was most closely correlated with subsequent rate of eGFR decline.

**Table 7:** Multiple linear regression models to determine the relationships between age, blood leukocyte, and monocyte subtypes, and rate of renal functional decline in study subjects with CKD 1–5.

**Model**	**Cohort**	**Number**	**Response variable**	**Explanatory variable**	**Coefficient**	**95% CI**	**^†^*P***
5	CKD 1-5	135	Rate of eGFR decline	Constant	0.00629	−0.00377, 0.01635	0.22
				Age (years)	−0.00284	−0.00560, −0.00007	0.04
				^#^Monocytes	−0.00098	−0.00313, 0.00117	0.37
				^#^Lymphocytes	−0.00084	−0.00352, 0.00184	0.54
				^#^Neutrophils	0.00022	−0.00203, 0.00247	0.85
6	CKD 1-5	124	Rate of eGFR decline	Constant	0.00536	−0.0033, 0.01401	0.22
				Age (years)	−0.00247	−0.00536, 0.00042	0.09
				^#^Classical	−0.00042	−0.00278, 0.00193	0.72
				^#^Intermediate	−0.00122	−0.00341, 0.00097	0.27
				^#^Non-classical	−0.00077	−0.00364, 0.00211	0.60
7	CKD 1-5	124	Rate of eGFR decline	Constant	0.00378	−0.00456, 0.01211	0.37
				Age (years)	−0.00185	−0.00466, 0.00095	0.19
				^#^Classical	0.00075	−0.00163, 0.00313	0.53
				^#^DR^mid^ Int.	0.00239	−0.00037, 0.00516	0.09
				^#^**DR**^hi^ **Int**.	−0.00664	−0.01044, −0.00283	**0.001**
				^#^Non-classical	0.00138	−0.0017, 0.00447	0.38

† *Statistical test = Multiple linear Regression Model with age correction*.

# *Represented as cells/mL. Cell types for which significant results were observed are bolded for emphasis*.

## Discussion

In 2013, 2.2 million deaths and 52 million disability-adjusted life years (DALYs) were associated with CKD, accounting for 3.9% of total global deaths and 2.1% of total global DALYs that year (39). This highlights the importance of targeting declining renal function and the critical nature of early diagnoses. In keeping with published studies, the work presented here has demonstrated that the absolute numbers of neutrophils and monocytes in venous blood are higher in adults with clinically-diagnosed CKD than in healthy adults without CKD (15, 19, 30, 32, 40, 41). Furthermore, peripheral blood counts for these two myeloid lineage cell types correlated with renal function (as reflected in creatinine-based eGFR) independently of age and gender. In more detailed analyses of the monocyte repertoire, however, we observed that a subpopulation of intermediate monocytes with higher HLA-DR surface expression correlated most strongly with eGFR and that only this subpopulation demonstrated a significant association with prospectively-quantified rate of eGFR decline. From the perspective of clinical relevance, our outpatient cohort encompassed a diverse range of underlying causes of CKD, did not include individuals with acute illness or hematological conditions and was under active management by consultant nephrologists at a tertiary referral center.

### Blood Monocyte Abnormalities Are Linked to CKD and Its Adverse Outcomes

Micro-inflammation, including dysregulation of circulating myeloid cells, contributes to the cardiovascular disease burden associated with CKD (15, 19, 30, 32, 40, 41). Abnormalities of the circulating monocyte repertoire may specifically contribute to the escalation of cardiovascular risk that occurs as CKD progresses by driving endothelial dysfunction and atherosclerosis (42). Indeed, blood monocyte number has been known for some time to have general predictive value for ASCVD (43). For example, Prentice et al. (44) observed that increased numbers of blood neutrophils and monocytes were associated with coronary artery disease. Additionally, in a report from the Atherosclerosis Risk in Communities (ARIC) study, the highest quartile for blood monocyte count had 40% increased cardiovascular mortality compared to the lowest quartile (45). Furthermore, total monocyte count, monocyte-to-lymphocyte ratio and monocyte-to-high density lipoprotein ratio have variously been reported to have prognostic value for ASCVD outcomes (43, 45–47). Most strikingly, in a large epidemiological study in the US, Bowe et al. recently demonstrated a linear relationship between blood monocyte count and adverse renal outcomes including doubling of serum creatinine, eGFR decline ≥30%, or a composite of ESRD, dialysis, or renal transplantation (19).

Abnormalities of monocyte function have also been shown to play an important pathophysiological role in CKD/ESRD. Dysregulated monocyte secretion of pro-inflammatory (TNFα and IL-6) and anti-inflammatory (IL-10) cytokines has long been reported under uremic conditions, particularly in the setting of dialysis-dependent ESRD (48, 49). Impaired recognition of pathogen-associated molecular patterns (PAMPs) to active innate immune responses, reduced expression of co-stimulatory molecules (CD80, CD86) and reduced antigen presenting capability, have also been reported to contribute to uremic immune dysfunction (48, 50). Additionally, hyperphosphatemia in CKD/ESRD may drive monocyte differentiation to osteoclast-like cells as a mechanism of vascular calcification (51). Our observations regarding total monocyte and monocyte subpopulation numbers across a broad range of eGFR values provide further evidence that abnormalities of the blood monocyte repertoire represent both a readily-quantifiable biomarker of the severity of CKD, albeit of as-yet unclear clinical value for individual patients. Such associations also represent an important clue to the specific mechanisms that link chronic micro-inflammation to CKD progression and adverse cardiovascular outcomes.

### Intermediate Monocyte Numbers Are Most Closely Associated With Abnormal Renal Function

A number of studies have provided evidence for heterogeneity within the intermediate monocyte subset (36, 37, 52–54). We report here that the absolute circulating number of an intermediate monocyte subpopulation that we have previously reported to more closely resemble non-classical than classical monocytes (22) was specifically increased in adults with medically-managed CKD, was inversely correlated with eGFR across the range of non-dialysis-dependent CKD and was associated with subsequent rate of eGFR decline. Monocyte heterogeneity is now widely acknowledged and there is a growing body of evidence that CD16^+^ monocytes specifically contribute to atherosclerosis both in the general population and in adults with CKD/ESRD (15). In 1998, Nockher et al. reported increased CD16^+^ monocyte numbers in blood samples from adults undergoing hemodialysis (55). Although the three-subset convention had not been fully established at that time, this study did highlight the fact that there were interesting gaps in our knowledge regarding monocyte subset dynamics in CKD. Following this, a number of studies confirmed and extended the observation of higher proportions or absolute numbers of CD16^+^ monocyte subsets in blood samples from adults with dialysis-dependent ESRD as well as those with pre-dialysis CKD (28–33, 56).

With the recognition of a distinction between intermediate and non-classical monocytes in 2010, several research groups have focussed on identifying specific monocyte subsets that are expanded in CKD/ESRD and on determining their association with known ASCVD risk factors and adverse outcomes. Although there are important common findings among these studies, there are also unresolved questions—particularly in regard to whether dysregulation of intermediate or non-classical monocytes is most prominent in the setting of non-RRT-dependent CKD. For example, reports from Merino et al. and Lee et al. document proportionate expansion of non-classical monocytes (defined in these studies as CD14^+^CD16^+^) in groups of pre-dialysis CKD patients (28, 31). Furthermore, Lee et al. demonstrated that CD14^+^CD16^+^ monocytes correlated significantly with eGFR and with an index of arterial stiffness (31). In contrast, in multiple cross-sectional and prospective studies, Heine and colleagues have consistently observed that proportions and absolute numbers of intermediate monocytes (defined as CD14^++^CD16^+^) are increased in pre-dialysis CKD, while non-classical monocyte numbers are only increased in dialysis-requiring ESRD patients (29, 30, 32, 33, 56). Furthermore, this group has convincingly demonstrated that, in CKD, circulating intermediate monocyte number is associated with ASCVD events, overall survival and adverse lipid profiles (30). From a mechanistic perspective, they have provided evidence that intermediate monocytes have high avidity for modified LDL with low capacity for cholesterol efflux—consistent with a pro-atherogenic phenotype (32).

The apparent discrepancies in findings from different researchers related to intermediate and non-classical monocytes could be the result of technical details, as multiple gating strategies have been reported for distinguishing the three monocyte subsets—the boundary between intermediate and non-classical being particularly prone to variability (33). It is also possible the monocyte repertoire abnormality “evolves” to sequentially involve intermediate and non-classical monocytes as CKD worsens and progresses to ESRD. The results we report here add the further explanation that “non-classical-like” DR^hi^ intermediate monocytes are specifically increased in number across the stages of CKD and may represent a distinctive immune/inflammatory response to reduced kidney function. Of interest, Patel et al. recently provided compelling evidence in healthy human subjects that blood intermediate monocytes represent a stage in the sequential maturation of a small proportion of bone marrow-derived classical monocytes to non-classical monocytes (23). Given the limited amount of knowledge regarding the mediators and signals that govern the transition of circulating monocytes from classical to intermediate to non-classical phenotypes, it is clear that CKD represents an important disease setting in which to study monocyte heterogeneity.

### The Link Between HLA-DR^hi^ Intermediate Monocyte Number and CKD Progression

As already noted, Bowe et al. observed at an epidemiological level that blood monocyte count has a linear relationship with clinically significant loss of renal function (19). While multiple studies have demonstrated that blood intermediate and/or non-classical monocyte numbers associate with ASCVD or atherogenic potential in patients with CKD/ESRD (28–32), the link between monocyte repertoire abnormalities and progression of CKD has been less investigated. In a subset of our well-characterized CKD 1–5 cohort with sufficient follow-up information, we identified a numerical expansion of DR^hi^ intermediate monocytes as being most closely correlated with rate of eGFR decline independently of age and gender. Although the finding will require further validation, it raises the possibility that this monocyte subpopulation or the factors that drive its increasing number in CKD have a specific role to play in the progression of chronic renal injury regardless of the primary cause for kidney disease. The pathophysiological roles of intermediate monocytes remain poorly understood although we and others have observed expansion of this subset in diverse pro-inflammatory conditions beyond CKD, including rheumatoid arthritis, inflammatory bowel disease and obesity (22, 57, 58). Advancement of knowledge in this area has been hampered by a number of factors. These include the lack of an intermediate monocyte subset in experimental rodent species, the complex and (until recently) contentious differentiation/maturation pathways of the three recognized subsets, the differential patterns of trans-endothelial migration and reverse migration of the monocyte subsets and accumulating evidence of further heterogeneity within the intermediate subset (36, 37, 52–54). Nonetheless, it is plausible that DR^hi^ intermediate monocytes mediate harmful effects on kidney structure through maladaptive interactions with the endothelium of intra-renal vasculature, by directly driving pro-fibrotic tubulointerstitial inflammation or by orchestrating the recruitment/transmigration of additional immune effectors from the blood to the kidney (59).

### Clinical Relevance and Conclusion

Our study adds to the evidence of clinical value in profiling blood intermediate monocytes in adults with CKD. Specifically, it will be of interest to determine whether enumeration of DR^hi^ intermediate monocytes in blood can be utilized in a specialist CKD clinic setting for risk stratification, monitoring of response to therapy or identification of target populations for clinical trials of novel agents. To better address the potential clinical applications, it will first be necessary to validate the associations in larger and more diverse patient cohorts and to perform longitudinal analyses involving patients progressing through the CKD stages. It will also be important to determine whether the associations between circulating monocytes and renal function are modified by specific clinical variables. In this regard, exploratory statistical analyses of the data for this study did not reveal significant interactions between diabetes, cardiovascular disease, proteinuria, renin angiotensin system inhibitor therapy or statin therapy, and the correlations between monocyte counts and eGFR (data not shown). However, adequately powered, targeted studies will be required to definitively answer these questions.

To develop targeted therapies, the pathophysiological mechanisms by which intermediate monocyte subsets contribute to loss of renal function need to be better understood. Infiltration of renal tissue and transformation to macrophages is a potential mechanism as macrophages have been implicated in the progression of renal fibrosis in a number of models (60–62). It has also been shown that renal macrophage number correlates with degree of renal fibrosis in human kidney biopsies (63). Further work characterizing monocyte and macrophage number and phenotype in tissue samples from individuals with CKD would be of great value. From the perspective of pathophysiological relevance, we foresee that purification, functional analysis, and molecular profiling of the intermediate monocyte sub-populations we describe here will have the potential to yield new insights into micro-inflammation associated with CKD and its major complications. In regard to neutrophil and lymphocyte abnormalities in CKD, it must be acknowledged that the flow cytometry-based definitions we used for these cells types were relatively crude. These analyses were designed to generate a high-level profile of the relative numbers of each of the major blood leukocyte clusters in the absence and presence of CKD leading to a more detailed study of monocyte subpopulations. More precise definition and further refinement of subpopulations of neutrophils and lymphocytes would require additional staining panels encompassing multiple specific markers and will be the subject of future studies. Finally, analysis of renal biopsy specimens using emerging cytometry and imaging techniques (64) will bring the potential for understanding the extent to which specific blood monocyte subpopulations infiltrate the diseased kidney and contribute to progression of injury.

In conclusion, our results further highlight the importance of monocyte biology and heterogeneity in human CKD, particularly in the context of renal functional decline. While the precise mechanisms underlying the numerical expansion of DR^hi^ intermediate monocytes remain to be elucidated, we provide novel evidence that this circulating monocyte subpopulation is distinctly altered in patients with medically-managed CKD and merits further investigation for its clinical diagnostic/prognostic value and as well as for its mechanistic and therapeutic implications.

## Author Contributions

SN, SC, and TG were responsible for the conception of the work, experimental design, acquisition, analysis and interpretation of the data, writing and final approval of the manuscript. SM, WM, JF, DC, and EC contributed to experimental design, data analysis and interpretation and writing and final approval of the manuscript. MD contributed to the conception of the work, interpretation of the data and the writing and final approval of the manuscript. MG was responsible for the conception, funding and supervision of the work, contributed to the experimental design, interpretation of the data, writing and final approval of the manuscript.

### Conflict of Interest Statement

The authors declare that the research was conducted in the absence of any commercial or financial relationships that could be construed as a potential conflict of interest.
